# Relationships of the competitor, stress tolerator, ruderal functional strategies of grass species with lifespan, photosynthetic type, naturalization and climate

**DOI:** 10.1093/aobpla/plad021

**Published:** 2023-04-29

**Authors:** Astrid Wingler, Brody Sandel

**Affiliations:** School of Biological, Earth & Environmental Sciences and Environmental Research Institute, University College Cork, Cork, Ireland; Department of Biology, Santa Clara University, Santa Clara, CA, USA

**Keywords:** annual, competitor, functional traits, grass family (Poaceae), introduced, leaf economics, native, perennial, ruderal, stress tolerator

## Abstract

Grass species (family Poaceae) are globally distributed, adapted to a wide range of climates and express a diversity of functional strategies. We explored the functional strategies of grass species using the competitor, stress tolerator, ruderal (CSR) system and asked how a species’ strategy relates to its functional traits, climatic distribution and propensity to become naturalized outside its native range. We used a global set of trait data for grass species to classify functional strategies according to the CSR system based on leaf traits. Differences in strategies in relation to lifespan (annual or perennial), photosynthetic type (C_3_ or C_4_), or naturalisation (native or introduced) were investigated. In addition, correlations with traits not included in the CSR classification were analyzed, and a model was fitted to predict a species’ average mean annual temperature and annual precipitation across its range as a function of CSR scores. Values for competitiveness were higher in C_4_ species than in C_3_ species, values for stress tolerance were higher in perennials than in annuals, and introduced species had more pronounced competitive-ruderal strategies than native species. Relationships between the CSR classification, based on leaf traits, and other functional traits were analyzed. Competitiveness was positively correlated with height, while ruderality was correlated with specific root length, indicating that both above- and belowground traits underlying leaf and root economics contribute to realized CSR strategies. Further, relationships between climate and CSR classification showed that species with competitive strategies were more common in warm climates and at high precipitation, whereas species with stress tolerance strategies were more common in cold climates and at low precipitation. The findings presented here demonstrate that CSR classification of functional strategies based on leaf traits matches expectations for the adaptations of grass species that underlie lifespan, photosynthetic type, naturalization and climate.

## Teaser

The family of grass species includes economically important food and forage crops. In addition, grasslands contribute to ecosystem services, such as carbon sequestration. This investigation shows that grass species have evolved different strategies that enable them to grow under a wide range of climatic conditions globally, and that these strategies can be determined from leaf traits: Slow-growing, stress-adapted grass species with long-lived leaves inhabit colder climates, whereas fast-growing, competitive species with large leaves grow in warmer climates. Climate change may contribute to the spreading of such competitive species into previously colder climates where they may outcompete native species.

## Introduction

The grass family (Poaceae), which includes about 12 000 species (Christenhusz and Byng 2016), is the economically most important plant family. The global distribution of grasses and their success in different climates depends on variation in functional traits, including traits that determine stress tolerance and phenology (Ocheltree *et al.* 2020; Schubert 2020). Frequent transitions from perennial to annual life cycles during grass evolution are associated with a higher allocation of biomass above ground (Lindberg *et al.* 2020). In addition, adaptation to the growth environment is reflected in photosynthetic type, with over 20 evolutionary C_3_/C_4_ transitions (Aliscioni *et al.* 2012).

Co-variation of leaf traits underlying a spectrum of strategies from slow to fast return of investment was described for global trait relationships in plants: the worldwide leaf economics spectrum (Wright *et al.* 2004) relates leaf longevity to high leaf mass per area (LMA) and low nitrogen content on a leaf mass basis (N_mass_). High LMA is generally associated with a conservative (slow) strategy, whereas high specific leaf area (SLA; the inverse of LMA) and N_mass_ are indicative of an acquisitive (fast) strategy. Although the leaf economics spectrum was originally used to describe covariation of traits for a wide range of plant growth forms in different biomes, it also applies to more closely related species, including the grass family. Analyzing trait relationships for a global set of grass species, Sandel *et al.* (2016) demonstrated correlations among leaf economics traits (SLA, N_mass_ and P_mass_) and among size-related traits (plant height, seed mass, leaf size and rooting depth). Similarly, a positive relationship between size-related traits (plant height and leaf area) was described for grass herbarium specimens (Jardine *et al.* 2020). Leaf area and height were also positively correlated not just among different grass species, but also within species sampled at different locations across California (Sandel *et al.* 2021). In addition, intra-specific co-variation patterns were observed in homogenous environments and common gardens, including negative correlations between SLA and leaf dry matter content (LDMC), and between LDMC and N_mass_ (Gorné *et al.* 2022), showing that trait variation patterns are not only driven by the immediate growth environment.

Co-variation in plant traits along axes of specialization can be used to identify plant functional strategies (Grime *et al.* 1997). This classification of functional strategies is based on adaptations to stress and/or disturbance. According to Grime (1977), stress refers to environmental constraints, such as a shortage or excess of water, light or mineral nutrients, sub- or supra-optimal temperatures, as well as toxins and pollutants. Disturbance, in this scheme, is defined as destruction of vegetation by humans, herbivores or natural catastrophes. Three primary strategies are described by Grime (1977): competitors (C) are adapted to low stress and low disturbance, stress tolerators (S) to high stress and low disturbance, and ruderals (R) to low stress and high disturbance. Annuals are expected to have predominantly ruderal strategies, whereas stress-tolerance strategies are common in long-lived perennials (Grime 1977). Classification of the CSR strategies of 30 grass species growing in northern Italy showed that competitive/ruderal strategies dominated in lowland species, whereas alpine species had stress-tolerator strategies (Pierce *et al.* 2007).

Building on CSR theory and the leaf economics spectrum, Pierce *et al.* (2013, 2017) developed the StrateFy tool to ascribe CSR functional strategies based on the following three leaf traits: SLA, LDMC and leaf area (LA). While the trade-off between SLA and LDMC reflects the leaf economics spectrum, LA represents the spectrum of plant size, a principal component perpendicular to leaf economics (Pierce *et al.* 2013; Díaz *et al.* 2016). Application to species of the grass genus *Poa* confirmed ruderal strategies for lowland grass species and stress tolerator strategies for montane species (Pierce *et al.* 2013). Recently, Yu *et al.* (2022) used the StrateFy tool for alpine Tibetan grasslands, demonstrating that stress tolerator strategies dominated. Using the same method, intrageneric and intraspecific variation was found for the grass genus *Brachypodium*, showing variation along the S and R axes in perennial *Brachypodium* species, while the annual model species *B. distachyon* was classified to have a less competitive strategy (Crowley and Wingler 2020). Intraspecific variation in functional strategies was also described for perennial ryegrass (*Lolium perenne*), demonstrating a trade-off between growth and dehydration survival (Keep *et al.* 2021). Such variation is reflected in CSR classification, which showed that perennial ryegrass has a mainly ruderal strategy, but with variation along the S- and R-axes (Crowley and Wingler 2020).

Although traits may be expected to vary in response to climate, global climate-trait relationships for different grass species are mainly weak (Jardine *et al.* 2020); however, culm length was positively correlated with mean annual precipitation (Sandel *et al.* 2016). While trait variation in the global context may be confounded by substantial variation within grass biomes (Jardine *et al.* 2020), clearer climate-trait relationships were detected in California, where a positive relationship between SLA and temperature was demonstrated (Sandel *et al.* 2021).

Compared to native plant species, introduced and particularly invasive species are overall larger with higher SLA, and more but smaller seeds (Ordonez *et al.* 2010; van Kleunen *et al.* 2010), suggesting that they are strong competitors, but also have traits characteristic of ruderal plants. Higher values of traits such as SLA, photosynthesis and N_mass_ indicate an acquisitive strategy. While trait relationships are the same for invasive and native species at the global level, the patterns differed when data were analyzed at the regional level, suggesting that different plant life forms may drive global patterns (Funk *et al.* 2017). For introduced grass species, higher SLA than for natives was described in California (Sandel and Low 2019) and globally (Broadbent *et al.* 2020; Monnet *et al.* 2020). There is also a general trend for introduced, and in particular naturalized grass species to grow taller than native species (Visser *et al.* 2016; Monnet *et al.* 2020), indicating higher competitive ability. However, while introduced species in California had larger seeds than native species (Sandel and Low 2019), seeds of naturalized species were smaller than those of native species in a global analysis (Monnet *et al.* 2020). In addition, naturalized species are more likely to be annuals with C_4_ photosynthesis (Monnet *et al.* 2020).

The aim of this study was to investigate the relationship between CSR functional strategies (according to Pierce *et al.* 2013, 2017) and other traits using a set of 465 grass species. We tested the following hypotheses: (i) CSR strategies identified based on leaf traits reflect other functional traits such as plant height and root traits. (ii) Strategies differ according to lifespan (annual/perennial), photosynthetic type (C_3_/C_4_), and naturalization (introduced/native). Specifically, we expected perennial grass species to have more pronounced stress tolerance strategies than annuals, C_4_ species to be stronger competitors than C_3_ species, and introduced species to have competitive/ruderal strategies. (iii) CSR strategies reflect adaptations to climate, such as species with C strategies growing in more favourable climates than those with S strategies.

## Materials and Methods

### Dataset

A global grass trait database (Sandel *et al.* 2016; Griffin-Nolan and Sandel in review) was used for trait data sources **[see****Supporting Information—References****]**. The dataset was filtered for species for which the following three leaf traits were available: LA, SLA and LDMC. This resulted in a dataset of 465 grass species **[see****Supporting Information—Table S1****]**, with a bias towards the northern hemisphere **[see****Supporting Information—Figure S1****]**. The dataset included 278 species of the BOP clade (271 species in the subfamily Pooideae, 6 in the Bambusoideae, and 1 in the Oryzoideae) and 187 of the PACMAD clade (92 species in the subfamily Panicoideae, 68 in the Chloridoideae, 16 in the Arundinoideae, and 11 in the Aristidoideae). The species were further classified according to lifespan (annual or perennial), photosynthetic type (C_3_ or C_4_), or naturalization. Photosynthetic type (C_3_ or C_4_) was largely assigned using the database published by Osborne *et al.* (2014). For three species photosynthetic type could not be ascertained. To identify differences related to naturalization status, the species were divided into those that grow only in their native environment (native) and those that have naturalized beyond their native environment (introduced), as classified by Monnet *et al.* (2020)(https://datadryad.org/stash/dataset/doi:10.5061/dryad.bvq83bk63) using the World Checklist of Selected Plant Families (WCSP; Royal Botanic Gardens Kew 2015). In cases where different species names were used in the original dataset and by Monnet *et al.* (2020), synonyms were matched using the Plants of the World Online database (http://www.plantsoftheworldonline.org/).

### Data analysis

CSR classification was conducted with the Microsoft Excel-based StrateFy tool (Pierce *et al.* 2017) using the following leaf traits: leaf area (LA; mm^2^), leaf dry matter content (LDMC; %) and specific leaf area (SLA; mm^2^ mg^−1^). Outputs from the tool were used to create ternary plots using SigmaPlot (Systat Software Inc.). The method for allocating CSR strategies was developed by Pierce *et al.* (2013) by translating the axes from a principal component analysis (PCA) into a CSR triangle. Specifically, two axes of variation were identified in the PCA: The first axis describes the range from acquisitive (high SLA, high N_mass_) to conservative (high LDMC, high leaf carbon content) leaf economic trait values. The second, perpendicular axis reflects the size spectrum (log LA and log leaf dry weight). Regression analysis was then used to develop the equations that describe the relationships between LA, LDMC and SLA with the two main PCA coordinates (Pierce *et al.* 2013).

All further analyses were conducted in R (R Core Team 2022). Testing for normality using the Shapiro-Wilk test showed that C, S and R data were not normally distributed. Non-parametric tests were used throughout: The Wilcoxon rank sum test was used to compare C, S and R values between annuals and perennials, between C_3_ and C_4_ species, and between native and introduced species; the Kruskal–Wallis rank sum test was used to compare C, S and R values among grass subfamilies; and the Spearman rank test was used to analyze correlations between C, S and R values and other traits.

The relationship between climate and a species’ C, S and R values was examined. For each species its distributional data at Biodiversity Information Standards (TDWG) level 3 (countries or states/provinces within large countries) was obtained from the World Checklist of Selected Plant Families from the Royal Botanical Gardens, Kew (http://wcsp.science.kew.org/), and the average mean annual temperature and annual precipitation were computed across a species’ entire range. Using all species, a linear model was then fitted to predict the mean annual temperature or annual precipitation value for a species as a function of its C, S and R scores, photosynthetic pathway, and lifespan, including all pairwise interactions except the photosynthetic pathway-by-lifespan interaction.

## Results

### CSR functional strategies dependent on lifespan, photosynthesis and naturalization

**Figure 1.**
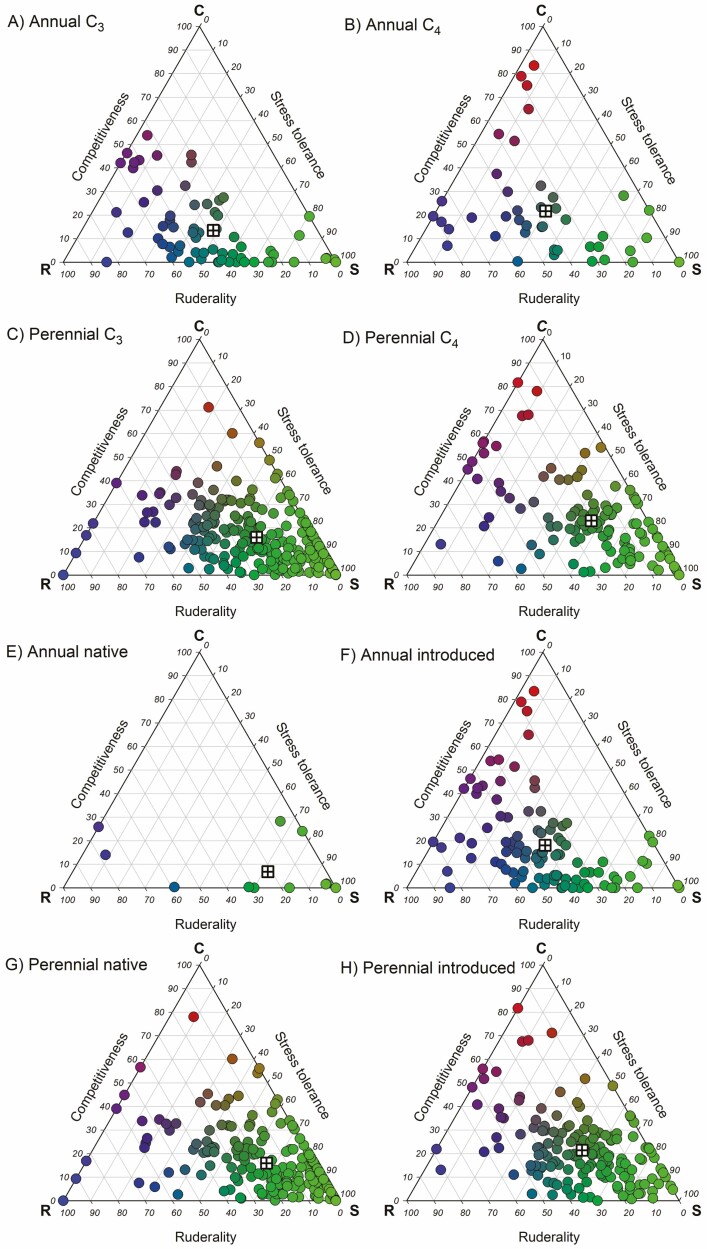
Triangulation of CSR functional strategies in grass species expressed as percent competitiveness, stress tolerance and ruderality, as calculated with the StrateFy tool (Pierce *et al.* 2017). Species were classified according to lifespan (annual or perennial), photosynthetic type (C_3_ or C_4_), or naturalization, i.e. species that grow only in their native environment (native) and those that have naturalized beyond their native environment (introduced), A. Annual C_3_ species; B. Annual C_4_ species; C. Perennial C_3_ species; D. Perennial C_4_ species; E. Annual native species; F. Annual introduced species; F. Perennial native species; H. Perennial introduced species. Individual species are represented as circles; the mean for the species in each plot is shown as crossed square.

CSR classification was performed using the StrateFy tool based on leaf data, giving the relative proportions of C, S and R components for each species (Pierce *et al.* 2017). Before plotting, the species were divided into annuals and perennials and then further subdivided into C_3_ and C_4_ species, and species that have naturalized outside their native range (introduced) and species only occurring in their native range (native) (Fig. 1A–H).

**Figure 2.**
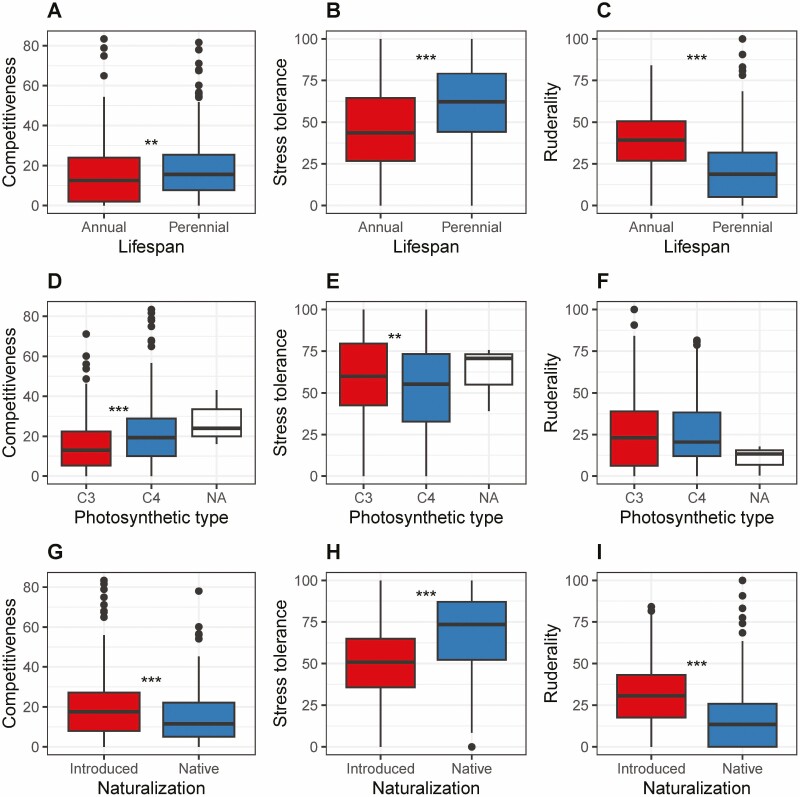
Comparison of CSR strategies between different functional groups of grass species; annual vs. perennial species (‘Lifespan’; A, B, C); C_3_ vs. C_4_ species (‘Photosynthetic type’; D, E, F; ‘NA’ = not available); introduced vs. native species (‘Naturalization’; G, H, I). Comparisons are shown for competitiveness (panels A, D, G), stress tolerance (panels B, E, H) and ruderality (panels C, F, I). Horizontal lines in the boxplots represent medians, boxes quartiles, whiskers the minimum/maximum and circles outliers. Asterisks indicate statistically significant differences (Wilcoxon rank sum test); * *P* < 0.05, ** *P* < 0.01, *** *P* < 0.001.

There were no annual C_3_ species with high values for C, suggesting no strong competitive strategy (Fig. 1A), but a small number of annual C_4_ species had high C values (Fig. 1B). Similarly, for the perennial species, high C values were mainly found among the C_4_ species (Fig. 1C and D). On average (C_3_ and C_4_ species combined), perennials had only slightly higher C values (*P* = 0.008; Wilcoxon rank sum test) than annuals, but more significantly higher values for S (*P* = 1.12 × 10^−7^), while values for R were lower in perennials than annuals (*P* = 4.30 × 10^−15^) (Fig. 2A–C). This suggests stronger stress tolerance strategies and less ruderality in the perennials than the annuals. On average (annuals and perennials combined), C_4_ species had higher values for C (*P* = 1.68 × 10^−5^) and lower values for S (*P* = 0.009) than C_3_ species, in agreement with a higher competitive ability of C_4_ species. However, values for R were not different between C_3_ and C_4_ species (Fig. 2D–F).

Only a few of the annual species were not introduced somewhere, and these species had low C-values (Fig. 1E). On average, introduced species had higher C values (*P* = 5.38 × 10^−4^), higher R values (*P* = 2.69 × 10^−15^) and lower S values (*P* = 1.41 × 10^−14^) than native species (Fig. 2G–I), suggesting stronger competitive and ruderal strategies, but less investment in stress tolerance.

CRS strategies were affected by taxonomic classification, as indicated by the effect of subfamily of the grass species on the values of C (*P* = 2.30 × 10^−12^; Kruskal-Wallis rank sum test), S (*P* = 3.79 × 10^−11^) and R (*P* = 3.80 × 10^−5^). For example, the Panicoideae, which include C_4_ crops such *Zea mays* and *Sorghum bicolor*, had higher C values (*P* = 1.20 × 10^−12^; Dunn test with Holm adjustment) and lower S values (*P* = 3.91 × 10^−8^) than the Pooideae, cool-season grasses which are exclusively C_3_.

### Relationship between CSR functional strategies and other traits

**Figure 3.**
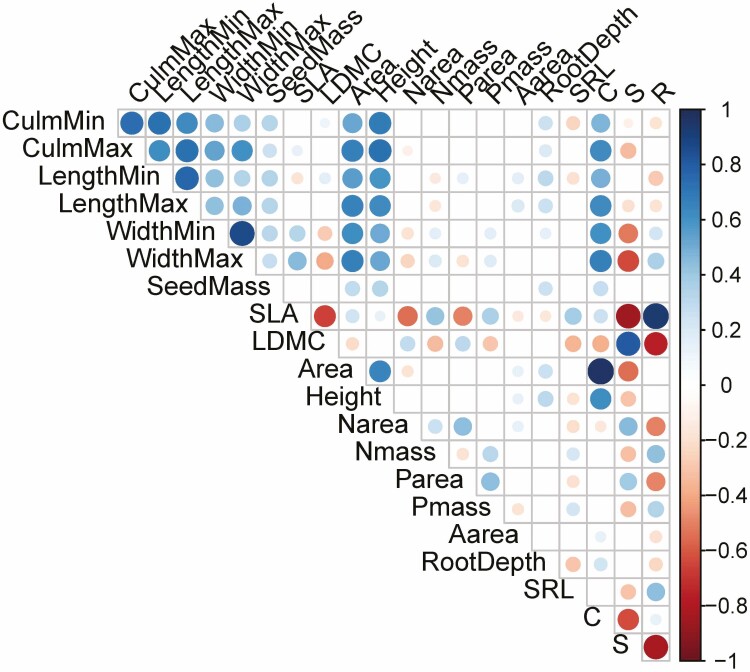
Correlation between CSR function strategies (C, S, R). Colour and size of the circles represent *rho*-values for significant correlations (*P* ≤ 0.05; Spearman rank test). CulmMin = minimum culm length; CulmMax = maximum culm length; LengthMin = minimum leaf length; LengthMax = maximum leaf length; WidthMin = minimum leaf width; Width Max = maximum leaf width; SeedMass = seed mass; SLA = specific leaf area; LDMC = leaf dry matter content; Area = leaf area; Height = plant height; N_area_ = nitrogen content on leaf area basis; N_mass_ = nitrogen content on leaf mass basis; P_area_ = phosphorus content on leaf area basis; P_mass_ = phosphorus content on leaf mass basis; A_area_ = photosynthesis on leaf area basis; RootDepth = root depth; SRL = specific root length; C = competitiveness; S = stress tolerance; R = ruderality. The number of observations, *P*-values and *rho*-values are given in **[see****Supporting Information—Table S2****]**.

**Figure 4.**
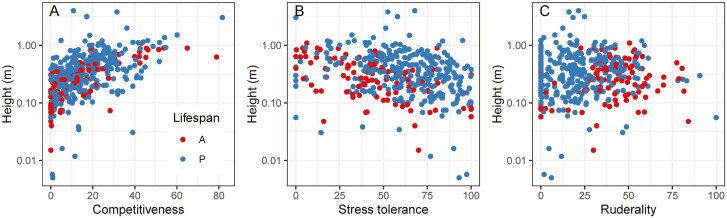
Relationship between CSR strategies and plant height of grass species; competitiveness (A), stress tolerance (B) and ruderality (C). Red circles = annuals (A); blue circles = perennials (P). Spearman rank correlations with height were analyzed. Competitiveness (% C) annuals: *P* < 0.001, *rho* = 0.794. Competitiveness (% C) perennials: *P* < 0.001, *rho* = 0.529. Stress tolerance (% S) annuals: *P* < 0.001, *rho* = −0.535. Stress tolerance (% S) perennials: *P* < 0.001, *rho* = −0.331. Ruderality (% R) annuals: *P* = 0.531, *rho* = 0.064. Ruderality (% R) perennials: *P* = 0.232, *rho* = 0.071.

Across all grass species, the values for C were positively correlated with size-related traits, including plant height (Fig. 3), with strong positive correlations of C and height both in annuals (*P* < 2.2 × 10^−16^, *rho* = 0.794) and perennials (*P* < 2.2 × 10^−16^, *rho* = 0.529) (Fig. 4A). Among the perennials, tall bamboo species were outliers with smaller than expected C values calculated from the leaf traits. In addition, values for C were positively correlated with seed mass and root depth, and more weakly with the rate of photosynthesis (A_area_). In addition, C values and height were positively correlated with seed mass.

Values for S were negatively correlated with size-related traits, including plant height for all species combined (Fig. 3), and when analyzed separately for annuals (*P* = 1.39 × 10^−8^, *rho* = −0.535) and perennials (*P* = 9.08 × 10^−9^, *rho* = −0.331) (Fig. 4B). In addition, the S values were positively correlated with N and P content on a leaf area basis, but negatively with N and P content on a leaf mass basis. There was also a negative correlation of S values with specific root length (SRL; i.e. root length divided by root dry mass).

Values for R were negatively correlated with N and P area, but positively with N and P mass, negatively with root depth, but positively with SRL (Fig. 3). In addition, SRL was positively correlated with SLA and negatively with LDMC, which underlies the positive correlation with R and the negative correlation with S. This would be expected if stress tolerators have longer-lived roots than ruderal species. No significant correlation of R values with height were found (Fig. 4C).

### Relationship between functional strategies and climate

**Figure 5.**
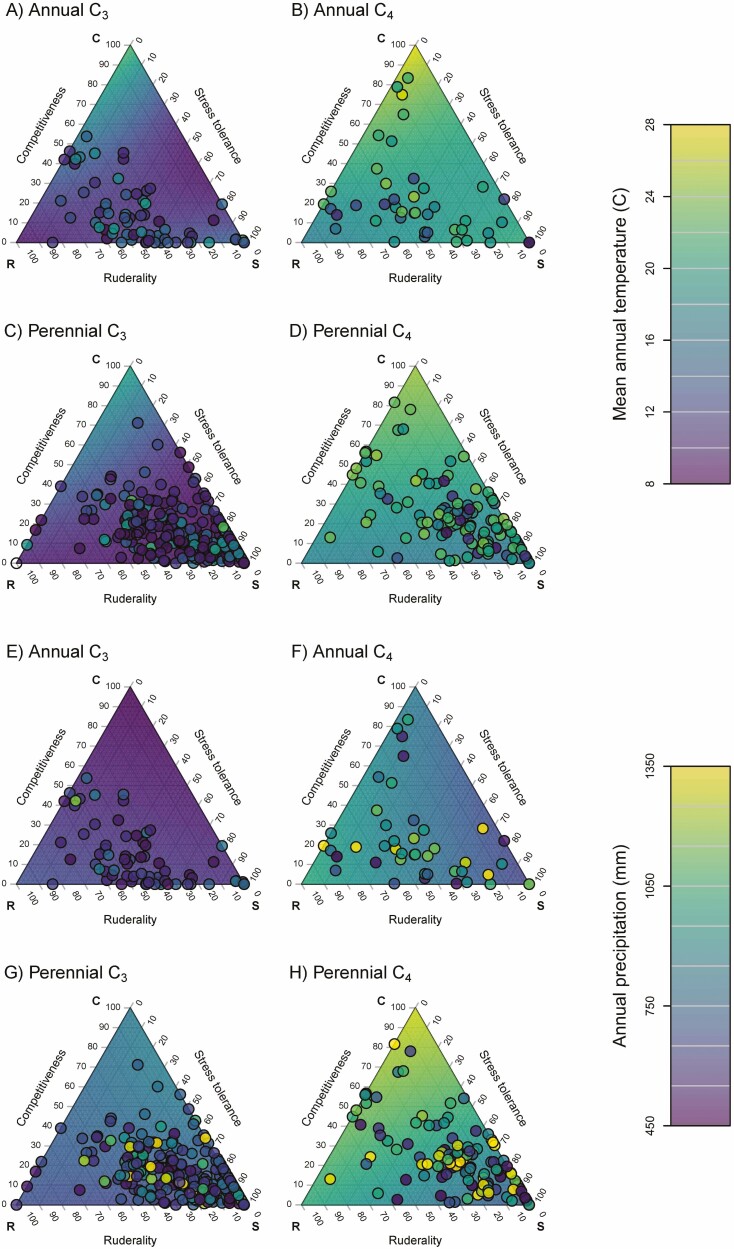
Predicted temperature (A–D) and annual precipitation (E–H) according to CSR functional strategy of grass species. Annual C_3_ species (A and E); annual C_4_ species (B and F); perennial C_3_ species (C and G); perennial C_4_ species (D and H). Symbols show the CSR positions of grass species, coloured according to the average mean annual temperature or annual precipitation within its range. The background colour indicates the predicted temperature or precipitation for a species at that position in each triangle.

The average mean annual temperature and annual precipitation were computed across each species’ range and plotted with the predicted climate variables from linear models within the CSR triangle (Fig. 5). This showed that C_3_ species tend to inhabit cooler (Fig. 5A and C), drier climates (Fig. 5E and G), and C_4_ species warmer (Fig. 5B and D), wetter climates (Fig. 5F and H). Perennials tend to grow in wetter climates (Fig. 5G and H) than annuals (Fig. 5E and F). Within these general patterns, species with high C values tend to occur in the warmest temperatures. Among C_3_ perennials, species with high R and S values are associated with relatively low annual precipitation, and species with high C values associated with high precipitation. Among C_4_ perennials, species with high C values were also associated with the highest precipitation levels, and those with high S values with the lowest precipitation.

## Discussion

Using trait data for 465 grass species with global distribution but bias towards origin from the northern hemisphere (especially northern Europe) **[see****Supporting Information—Figure S1****]**, we classified CSR functional strategies based on leaf data with the StrateFy tool (Pierce *et al.* 2017). CSR strategies matched expectations for lifespan, photosynthetic type and naturalization status (Fig. 1): perennial grass species had stronger stress tolerance strategies but lower ruderality than annuals; C_4_ species had strategies associated with stronger competitive ability than C_3_ species; and naturalized introduced species were characterized by more pronounced competitive/ruderal strategies than native species (Fig. 2).

Importantly, relationships with independent traits (not directly connected to the leaf traits used for the CSR classification) confirmed that CSR classification describes plant functional strategies beyond leaf economics. For example, plant height was positively correlated with the calculated values for C but negatively with S (Figs. 3 and 4), showing a trade-off between growth/competitiveness and stress tolerance. In addition, root depth was positively correlated with values for C but negatively correlated with R, while specific root length (SRL) emerged as a trait that may underlie the trade-off between stress tolerance and ruderality. Further, the analyses show that colder and drier climates are associated with higher S values and lower C values (Fig. 5), suggesting that climate determines the trade-off between stress tolerance and competitiveness.

### CSR classification of grass species reflects whole-plant strategies

Our results show that CSR classification based on leaf traits not only reflects leaf economics, but also wider, whole-plant strategies. Values for C were positively and S values negatively correlated not just with leaf size, but also other size-related traits, such as culm length and plant height (Figs. 3 and 4). This is in agreement with positive correlations between size-related traits in grass species (Sandel *et al.* 2016). These findings reflect a trade-off between investment of carbon in growth and in defense structures that are characteristic of long-lived leaves of stress tolerators, such as investment in sclerenchyma (Pierce *et al.* 2007).

Other schemes for classification of strategies highlight the importance of plant height. While Westoby (1998) developed the leaf-height-seed scheme on the assumption that trade-offs exist between these traits, Díaz *et al.* (2016) identified two main axes of trait variation, one describing leaf economics, the other linking plant height and diaspore size. Our analysis reveals positive correlations of seed mass with plant height, leaf area and C values, suggesting that there is no trade-off between different size-related traits among grass species. However, the results presented here are based on species averages and do not reflect intraspecific variation, which could result in trade-offs between investment in reproduction and growth, especially in low-resource environments.

The relationships between the allocated CSR strategies and nutrient contents, such as positive correlation of R values with N_mass_ and P_mass_, but negative correlation of S values with these parameters are based on leaf economics, reflecting the spectrum from acquisitive/short-lived leaves with high SLA and N_mass_ to conservative/long-lived leaves with low SLA and low N_mass_ (Wright *et al.* 2004; Díaz *et al.* 2016). Equally, positive correlations between SLA and N_mass_ were described for grass species (Sandel *et al.* 2016; Jardine *et al.* 2020).

### CSR classification connects leaf with root economics

As described previously (Sandel *et al.* 2016), root depth was positively correlated with size-related traits such as leaf size and plant height. Our results also reveal a positive correlation between root depth and competitiveness, but a negative correlation with ruderality (Fig. 3). While we would have expected stress tolerance strategies to be related to root depth, no such relationship was found. Instead, root depth was positively correlated with above-ground organ size and values for C. This suggests that root depth reflects the overall size of grass species and not a trade-off in shoot-root allocation associated with increased below-ground resource acquisition under stress conditions.

The relationships of traits and strategies with SRL revealed here are of particular significance: SRL was positively correlated with values for R, but negatively with values for S. Since SRL was also positively correlated with SLA, our analysis links leaf economics with root economics (Roumet *et al.* 2016; Weigelt *et al.* 2021). Roumet *et al.* (2016) found a positive correlation between root respiration and SRL in graminoids, suggesting that the same acquisitive-conservative (or fast-slow) gradient which applies to leaves is also valid for roots (i.e. the root economics spectrum). Since root traits are more difficult to analyse than leaf traits, it is promising that CSR classification based on leaf traits can provide information that is also relevant for investment in the root system.

### The stress tolerance strategy of perennial species is determined by functional traits

In line with expectations (Grime 1977; Pierce *et al*. 2017), perennials were shown to have higher values for S but lower values for R than annuals (Fig. 2). This was driven by lower SLA but higher LDMC in the perennials, which is in line with findings for congeneric annual and perennial grass species (Garnier *et al.* 1992). These traits of perennials were associated with higher growth rates of annual grass species (Garnier *et al.* 1992; Garnier and Laurent 1994). However, we found slightly higher values for C in perennial species. Despite the described differences, strategies were overall similar, with a SR/CSR strategy in annuals and an S/CSR strategy in perennials, which is not surprising given that only grass species and not different plant growth forms were compared.

The more competitive strategy in C_4_ than C_3_ species was expected; the only species with very high C values were C_4_ species (Fig. 1), including the annual crops *Eleusine coracana* (Chloridoideae) and *Zea mays* (Panicoideae), and the perennials *Pennisetum purpureum* and *Loudetia phragmitoides* (both Panicoideae). This also reflects the higher C values in the Panicoideae (mostly C_4_) than the Pooideae (exclusively C_3_). Liu *et al.* (2019) described interactions between life history and photosynthetic type showing that annuality/perenniality is the determinant explaining traits in subtropical grasses, in line with the differences in functional strategy described here.

### Competitiveness and ruderality are associated with naturalization of introduced species

Introduced species had, on average, more competitive/ruderal strategies, but less pronounced stress tolerance strategies than native species (Fig. 2). This is in agreement with expectations based on trait comparisons between native and introduced grass species which show higher SLA (Sandel and Low 2019; Broadbent *et al.* 2020; Monnet *et al.* 2020), higher LA (Visser *et al.* 2016) and greater height (Visser *et al.* 2016; Monnet *et al.* 2020) for introduced species. Visser *et al.* (2016) ascribe the competitive success of invasive grasses in South Africa to this trait combination, but also highlight that the annual life cycle of invasive species points towards a ruderal strategy.

Tall grass species with greater than 2 m height are more likely to naturalize than shorter species, but probability of naturalization was only increased when bamboos and non-bamboos were analysed separately (Canavan *et al.* 2019). In our dataset, differences in height were only found when bamboos were excluded from the native species (no introduced bamboos were included in our analyses), with species averages of 42.8 cm for introduced species vs. 35.6 cm for native species. Importantly, we show that these trait differences underlying the naturalization success of introduced species can be captured in CSR functional strategies.

### Adaptation to climate underlie photosynthetic type, lifespan and functional strategy

The occurrence of C_4_ species in warmer climates (Fig. 5) was expected (Sage *et al.* 2012), whereas their occurrence in wetter climates was surprising since the evolution of C_4_ photosynthesis has been linked to reduced annual precipitation (Edwards and Smith 2010). C_4_ photosynthesis can operate at lower stomatal conductance than C_3_ photosynthesis which enables higher photosynthetic water-use efficiency and drier precipitation niches (Taylor *et al.* 2012). However, C_4_ species still benefit from high rainfall during the warm season (Zhou *et al.* 2018). In addition, tree cover and fire, which also determine C_4_ grass distribution (Griffiths *et al.* 2015), were not considered here.

Given the higher stress tolerance of perennial species and their more extensive root system, the association of perennial grass species with higher annual precipitation is surprising. However, seasonality of rainfall rather than total annual precipitation may be the critical factor because annual species can be better adapted to seasonally dry environments (Lindberg *et al.* 2020). Similarly, Liu *et al.* (2019) found that for subtropical grasses, annuals (especially C_4_ annuals) are distributed in regions with lower precipitation. The association of stress tolerance with low precipitation in our analysis (Fig. 5) can therefore not be explained with a perennial life history.

In agreement with the previous observation that culm length increased with precipitation (Sandel *et al.* 2016), the association of competitiveness with high precipitation shown here (Fig. 5) supports a more competitive strategy in wetter climates. Although stress tolerance strategies dominated in alpine grasslands on the Tibetan Plateau, the contribution of competitive and ruderal strategies increased with increasing precipitation (Yu *et al.* 2022). However, when grass species were analyzed separately, values for C were zero for all grasslands, and higher nutrient rather than water availability may explain the shift in strategy (Yu *et al.* 2022). Previously, Pierce *et al.* (2007) had also classified alpine grass species in Italy as stress tolerators, while lowland species had more pronounced competitive-ruderal strategies. While climate relationships were not analyzed by Pierce *et al.* (2007), adaptation to lower temperatures at higher elevation may have been the dominant factor underlying this difference.

Our analyses use species trait means and do not take intraspecific variation into account. For example, within Californian grass species, higher SLA and height were determined in warmer climates, and bias of available records can thus affect species means (Sandel *et al.* 2021). However, in the global context investigated here, which captures variation from arctic-alpine to tropical species, intraspecific variation is relatively less significant.

## Conclusions

We have shown that CSR functional strategies of grass species based on leaf economics reflect whole-plant strategies related to both above- and belowground resource acquisition. Perennial grass species had stronger stress tolerance and less pronounced ruderal strategies than annuals, C_4_ species had more competitive strategies than C_3_ species, and introduced species had more competitive-ruderal strategies than native species. Further, species with competitive strategies were more common in climates with favourable growth conditions, whereas those with stress tolerance strategies were more common in climates with low temperatures and low precipitation. Species distribution and invasion success are therefore affected by climate change, with competitive C_4_ invaders likely to become more successful as temperatures rise in currently colder climatic regions.

## Supporting Information

The following additional information is available in the online version of this article –

Supporting Information Figure S1. Geographic distribution of 465 grass species.

Supporting Information References. Trait data sources from TRY or the grass trait database.

Supporting Information Table S1. Trait data and CSR scores for 465 grass species.

Supporting Information Table S2. Spearman rank correlations among grass traits.

plad021_suppl_Supplementary_Material

plad021_suppl_Supplementary_Table_S1

plad021_suppl_Supplementary_Table_S2

plad021_suppl_Supplementary_Data

## Data Availability

Data are available as Supporting Information.

## Notes

**Populations & Communities. Guest editor: Jean Burns**
