# Supplementary material for: Relationships of the competitor, stress tolerator, ruderal functional strategies of grass species with lifespan, photosynthetic type, naturalization and climate
Source: AoB Plants. 2023 Apr 29;15(3):plad021. doi: 10.1093/aobpla/plad021 (PMC10184452; doi:10.1093/aobpla/plad021)
Supplement: plad021_suppl_Supplementary_Data [file plad021_suppl_supplementary_data.docx]

**References to trait data sources from TRY or the grass trait database**

Abbott, M.L., Fraley Jr, L. and Reynolds, T.D., 1991. Root profiles of selected cold desert shrubs and grasses in disturbed and undisturbed soils. Environmental and Experimental Botany, 31(2), pp.165-178.

Adler, P.B., Milchunas, D.G., Lauenroth, W.K., Sala, O.E. and Burke, I.C., 2004. Functional traits of graminoids in semi‐arid steppes: a test of grazing histories. Journal of Applied Ecology, 41(4), pp.653-663.

Adler, P.B., Salguero-Gómez, R., Compagnoni, A., Hsu, J.S., Ray-Mukherjee, J., Mbeau-Ache, C. and Franco, M., 2014. Functional traits explain variation in plant life history strategies. Proceedings of the National Academy of Sciences, 111(2), pp.740-745.

Al Haj Khaled, R., Duru, M., Theau, J.P., Plantureux, S. and Cruz, P., 2005. Variation in leaf traits through seasons and N‐availability levels and its consequences for ranking grassland species. Journal of Vegetation Science, 16(4), pp.391-398.

Albertson, F.W., 1937. Ecology of mixed prairie in west central Kansas. Ecological Monographs, 7(4), pp.481-547.

Atkin, O.K., Bloomfield, K.J., Reich, P.B., Tjoelker, M.G., Asner, G.P., Bonal, D., Bönisch, G., Bradford, M.G., Cernusak, L.A., Cosio, E.G. and Creek, D., 2015. Global variability in leaf respiration in relation to climate, plant functional types and leaf traits. New Phytologist, 206(2), pp.614-636.

Atkin, O.K., Westbeek, M.H., Cambridge, M.L., Lambers, H. and Pons, T.L., 1997. Leaf respiration in light and darkness (a comparison of slow-and fast-growing Poa species). Plant Physiology, 113(3), pp.961-965.

Atkinson, R.R., Mockford, E.J., Bennett, C., Christin, P.A., Spriggs, E.L., Freckleton, R.P., Thompson, K., Rees, M. and Osborne, C.P., 2016. C 4 photosynthesis boosts growth by altering physiology, allocation and size. Nature Plants, 2(5), pp.1-5.

Bai, W.M., Zhou, M., Fang, Y. and Zhang, W.H., 2017. Differences in spatial and temporal root lifespan of three Stipa grasslands in northern China. Biogeochemistry, 132(3), pp.293-306.

Bakker, L.M., Mommer, L. and van Ruijven, J., 2019. Using root traits to understand temporal changes in biodiversity effects in grassland mixtures. Oikos, 128(2), pp.208-220.

Baruch, Z. and Jackson, R.B., 2005. Responses of tropical native and invader C4 grasses to water stress, clipping and increased atmospheric CO_2_ concentration. *Oecologia*, *145*(4), pp.522-532.

Barkaoui, K., Roumet, C. and Volaire, F., 2016. Mean root trait more than root trait diversity determines drought resilience in native and cultivated Mediterranean grass mixtures. Agriculture, Ecosystems & Environment, 231, pp.122-132.

Baruch, Z. and Goldstein, G., 1999. Leaf construction cost, nutrient concentration, and net CO 2 assimilation of native and invasive species in Hawaii. Oecologia, 121(2), pp.183-192.

Batalha, M.A., Pipenbaher, N., Bakan, B., Kaligarič, M. and Škornik, S., 2015. Assessing community assembly along a successional gradient in the North Adriatic Karst with functional and phylogenetic distances. Oecologia, 178(4), pp.1205-1214.

Beaulieu, J.M., Leitch, I.J. and Knight, C.A., 2007. Genome size evolution in relation to leaf strategy and metabolic rates revisited. Annals of Botany, 99(3), pp.495-505.

Bergmann, J., Ryo, M., Prati, D., Hempel, S. and Rillig, M.C., 2017. Root traits are more than analogues of leaf traits: the case for diaspore mass. New Phytologist, 216(4), pp.1130-1139.

Birouste, M., Kazakou, E., Blanchard, A. and Roumet, C., 2011. Plant traits and decomposition: are the relationships for roots comparable to those for leaves?. Annals of Botany, 109(2), pp.463-472.

Blonder, B., Buzzard, V., Simova, I., Sloat, L., Boyle, B., Lipson, R., Aguilar‐Beaucage, B., Andrade, A., Barber, B., Barnes, C. and Bushey, D., 2012. The leaf‐area shrinkage effect can bias paleoclimate and ecology research. American Journal of Botany, 99(11), pp.1756-1763.

Blumenthal, Dana; Kray, Julie; Mueller, Kevin; Ocheltree, Troy (2020), Shortgrass steppe and northern mixedgrass prairie plant species traits, Dryad, Dataset, <https://doi.org/10.5061/dryad.8sf7m0cjr>

Bonham, C.D. and Mack, S.E., 1990. Root distributions of Eurotia lanata in association with two species of Agropyron on disturbed soils. Botanical gazette, 151(4), pp.522-527.

Bouchenak-Khelladi, Y., February, E.C., Verboom, G.A. and Boucher, F.C., 2020. C4 grass functional traits are correlated with biotic and abiotic gradients in an African savanna. *Plant Ecology*, *221*(4), pp.241-254.

Bragazza, L., 2009. Conservation priority of Italian Alpine habitats: a floristic approach based on potential distribution of vascular plant species. Biodiversity and conservation, 18(11), pp.2823-2835.

Brown, J. and J. Zinnert. 2020. Species composition and plant functional traits on Hog and Metompkin Islands, VA 2016-2017 ver 2. Environmental Data Initiative. https://doi.org/10.6073/pasta/11732b6818b08936b4b51a614c09c114 (Accessed 2022-05-22).

Brudvig, L.A. and Mabry, C.M., 2008. Trait‐based filtering of the regional species pool to guide understory plant reintroductions in Midwestern oak savannas, USA. Restoration Ecology, 16(2), pp.290-304.

Burrascano, S., Copiz, R., Del Vico, E., Fagiani, S., Giarrizzo, E., Mei, M., Mortelliti, A., Sabatini, F.M. and Blasi, C., 2015. Wild boar rooting intensity determines shifts in understorey composition and functional traits. Community ecology, 16(2), pp.244-253.

Burton, J.I., Perakis, S.S., McKenzie, S.C., Lawrence, C.E. and Puettmann, K.J., 2017. Intraspecific variability and reaction norms of forest understorey plant species traits. Functional Ecology, 31(10), pp.1881-1893.

Butterfield, B.J., Bradford, J.B., Munson, S.M. and Gremer, J.R., 2017. Aridity increases below-ground niche breadth in grass communities. Plant Ecology, 218(4), pp.385-394.

Cadotte, M.W., 2017. Functional traits explain ecosystem function through opposing mechanisms. Ecology Letters, 20(8), pp.989-996.

Campbell, C., Atkinson, L., Zaragoza‐Castells, J., Lundmark, M., Atkin, O. and Hurry, V., 2007. Acclimation of photosynthesis and respiration is asynchronous in response to changes in temperature regardless of plant functional group. New Phytologist, 176(2), pp.375-389.

Campetella, G., Botta-Dukát, Z., Wellstein, C., Canullo, R., Gatto, S., Chelli, S., Mucina, L. and Bartha, S., 2011. Patterns of plant trait–environment relationships along a forest succession chronosequence. Agriculture, ecosystems & environment, 145(1), pp.38-48.

Canadell, J., Jackson, R.B., Ehleringer, J.B., Mooney, H.A., Sala, O.E. and Schulze, E.D., 1996. Maximum rooting depth of vegetation types at the global scale. Oecologia, 108(4), pp.583-595.

Carmo‐Silva, A.E., Francisco, A., Powers, S.J., Keys, A.J., Ascensão, L., Parry, M.A. and Arrabaça, M.C., 2009. Grasses of different C4 subtypes reveal leaf traits related to drought tolerance in their natural habitats: changes in structure, water potential, and amino acid content. American Journal of Botany, 96(7), pp.1222-1235.

Carmona, C.P., Rota, C., Azcárate, F.M. and Peco, B., 2015. More for less: sampling strategies of plant functional traits across local environmental gradients. Functional Ecology, 29(4), pp.579-588.

Carrera, A.L., Mazzarino, M.J., Bertiller, M.B., del Valle, H.F. and Carretero, E.M., 2009. Plant impacts on nitrogen and carbon cycling in the Monte Phytogeographical Province, Argentina. Journal of Arid Environments, 73(2), pp.192-201.

Case, E.J., Harrison, S., Cornell, H.V. and Allen, E., 2016. Do high-impact invaders have the strongest negative effects on abundant and functionally similar resident species. Functional Ecology, 30(8), pp.1447-1453.

Chai, Y., Liu, X., Yue, M., Guo, J., Wang, M., Wan, P., Zhang, X. and Zhang, C., 2015. Leaf traits in dominant species from different secondary successional stages of deciduous forest on the Loess Plateau of northern China. Applied vegetation science, 18(1), pp.50-63.

Chandra, A. and Dubey, A., 2008. Evaluation of genus Cenchrus based on malondialdehyde, proline content, specific leaf area and carbon isotope discrimination for drought tolerance and divergence of species at DNA level. Acta Physiologiae Plantarum, 30(1), pp.53-61.

Chen, Y., Han, W., Tang, L., Tang, Z. and Fang, J., 2013. Leaf nitrogen and phosphorus concentrations of woody plants differ in responses to climate, soil and plant growth form. Ecography, 36(2), pp.178-184.

Cheng, J., Chu, P., Chen, D. and Bai, Y., 2016. Functional correlations between specific leaf area and specific root length along a regional environmental gradient in Inner Mongolia grasslands. Functional Ecology, 30(6), pp.985-997.

Choat, B., Jansen, S., Brodribb, T.J., Cochard, H., Delzon, S., Bhaskar, R., Bucci, S.J., Feild, T.S., Gleason, S.M., Hacke, U.G. and Jacobsen, A.L., 2012. Global convergence in the vulnerability of forests to drought. Nature, 491(7426), pp.752-755.

Ciccarelli, D., 2015. Mediterranean coastal dune vegetation: are disturbance and stress the key selective forces that drive the psammophilous succession?. Estuarine, Coastal and Shelf Science, 165, pp.247-253.

Ciocârlan, V., 2009. Illustrated flora of Romania. Pteridophyta et Spermatopyta (in Romanian). Editura Ceres.

Cochard, R. and Edwards, P.J., 2011. Tree dieback and regeneration in secondary Acacia zanzibarica woodlands on an abandoned cattle ranch in coastal Tanzania. *Journal of Vegetation Science*, *22*(3), pp.490-502.

Cooke, J. and Leishman, M.R., 2011. Silicon concentration and leaf longevity: is silicon a player in the leaf dry mass spectrum?. Functional Ecology, 25(6), pp.1181-1188.

Cornelissen, J.H.C., 1996. An experimental comparison of leaf decomposition rates in a wide range of temperate plant species and types. Journal of ecology, pp.573-582.

Cornelissen, J.H.C., Quested, H.M., GWYNN‐JONES, D., Van Logtestijn, R.S.P., De Beus, M.A.H., Kondratchuk, A., Callaghan, T.V. and Aerts, R., 2004. Leaf digestibility and litter decomposability are related in a wide range of subarctic plant species and types. Functional Ecology, 18(6), pp.779-786.

Costa, J.L. and Gomide, J.A., 1991. Drying rates of tropical grasses. Tropical Grasslands, 25, pp.325-332.

Craine, J.M., Elmore, A.J., Aidar, M.P., Bustamante, M., Dawson, T.E., Hobbie, E.A., Kahmen, A., Mack, M.C., McLauchlan, K.K., Michelsen, A. and Nardoto, G.B., 2009. Global patterns of foliar nitrogen isotopes and their relationships with climate, mycorrhizal fungi, foliar nutrient concentrations, and nitrogen availability. New Phytologist, 183(4), pp.980-992.

Craine, J.M., Lee, W.G., Bond, W.J., Williams, R.J. and Johnson, L.C., 2005. Environmental constraints on a global relationship among leaf and root traits of grasses. Ecology, 86(1), pp.12-19.

Craine, J.M., Nippert, J.B., Towne, E.G., Tucker, S., Kembel, S.W., Skibbe, A. and McLauchlan, K.K., 2011. Functional consequences of climate change-induced plant species loss in a tallgrass prairie. Oecologia, 165(4), pp.1109-1117.

Craine, J.M., Ocheltree, T.W., Nippert, J.B., Towne, E.G., Skibbe, A.M., Kembel, S.W. and Fargione, J.E., 2013. Global diversity of drought tolerance and grassland climate-change resilience. Nature Climate Change, 3(1), pp.63-67.

Craine, J.M., Tilman, D., Wedin, D., Reich, P., Tjoelker, M. and Knops, J., 2002. Functional traits, productivity and effects on nitrogen cycling of 33 grassland species. Functional Ecology, 16(5), pp.563-574.

Craine, J.M., Towne, E.G., Ocheltree, T.W. and Nippert, J.B., 2012. Community traitscape of foliar nitrogen isotopes reveals N availability patterns in a tallgrass prairie. Plant and soil, 356(1-2), pp.395-403.

Cruz, P., De Quadros, F.L.F., Theau, J.P., Frizzo, A., Jouany, C., Duru, M. and Carvalho, P.C.F., 2010. Leaf traits as functional descriptors of the intensity of continuous grazing in native grasslands in the south of Brazil. Rangeland Ecology & Management, 63(3), pp.350-358.

Cruz-Maldonado, N., Weemstra, M., Jiménez, L., Roumet, C., Angeles, G., Barois, I., de los Santos, M., Morales-Martinez, M.A., Palestina, R.A., Rey, H. and Sieron, K., 2021. Aboveground-trait variations in 11 (sub) alpine plants along a 1000-m elevation gradient in tropical Mexico. *Alpine Botany*, *131*(2), pp.187-200.

d'Amen, M., Mateo, R.G., Pottier, J., Thuiller, W., Maiorano, L., Pellissier, L., Ndiribe, C., Salamin, N. and Guisan, A., 2018. Improving spatial predictions of taxonomic, functional and phylogenetic diversity. Journal of Ecology, 106(1), pp.76-86.

Dalke, I.V., Novakovskiy, A.B., Maslova, S.P. and Dubrovskiy, Y.A., 2018. Morphological and functional traits of herbaceous plants with different functional types in the European Northeast. Plant Ecology, 219(11), pp.1295-1305.

de Vries, F.T. and Bardgett, R.D., 2016. Plant community controls on short‐term ecosystem nitrogen retention. New Phytologist, 210(3), pp.861-874.

Diaz, S., Hodgson, J.G., Thompson, K., Cabido, M., Cornelissen, J.H., Jalili, A., Montserrat‐Marti, G., Grime, J.P., Zarrinkamar, F., Asri, Y. and Band, S.R., 2004. The plant traits that drive ecosystems: evidence from three continents. Journal of vegetation science, 15(3), pp.295-304.

Dickie, J., 2008. Royal Botanical Gardens KEW. Seed Information Database (SID). Version 7.1.

Diemer, M., 1998. Life span and dynamics of leaves of herbaceous perennials in high‐elevation environments:‘news from the elephant’s leg’. Functional Ecology, 12(3), pp.413-425.

Dolezal, Jiri; Lanta, Vojtech; Mudrak, Ondrej; Leps, Jan (2019), Data from: Seasonality promotes grassland diversity: interactions with mowing, fertilization and removal of dominant species, Dryad, Dataset, <https://doi.org/10.5061/dryad.hm6nr2c>

Domingues, T.F., Martinelli, L.A. and Ehleringer, J.R., 2007. Ecophysiological traits of plant functional groups in forest and pasture ecosystems from eastern Amazonia, Brazil. Plant Ecology, 193(1), pp.101-112.

Dostál, Petr; Fischer, Markus; Chytrý, Milan; Prati, Daniel (2017), Data from: No evidence for larger leaf trait plasticity in ecological generalists compared to specialists, Dryad, Dataset, <https://doi.org/10.5061/dryad.p3057>

Drenovsky, R.E. and James, J.J., 2010. Designing invasion-resistant plant communities: the role of plant functional traits. Rangelands, 32(1), pp.32-37.

Dubey, A. and Chandra, A., 2008. Effect of water stress on carbon isotope discrimination and its relationship with transpiration efficiency and specific leaf area in Cenchrus species. Journal of environmental biology, 29(3), p.371.

Duru, M., Tallowin, J. and Cruz, P., 2005. Functional diversity in low-input grassland farming systems: characterisation, effect and management. Integrating efficient grassland farming and biodiversity’.(Eds R Lillak, R Viiralt, A Linke, V Geherman) pp, pp.199-210.

Dwyer, J.M., Hobbs, R.J. and Mayfield, M.M., 2014. Specific leaf area responses to environmental gradients through space and time. Ecology, 95(2), pp.399-410.

Everwand, G., Fry, E.L., Eggers, T. and Manning, P., 2014. Seasonal variation in the capacity for plant trait measures to predict grassland carbon and water fluxes. Ecosystems, 17(6), pp.1095-1108.

Fan, Y., Miguez-Macho, G., Jobbágy, E.G., Jackson, R.B. and Otero-Casal, C., 2017. Hydrologic regulation of plant rooting depth. Proceedings of the National Academy of Sciences, 114(40), pp.10572-10577.

Farkas, T.E., B.P. Gerstner, and K.D. Whitney. 2021. SEV-LTER Plant Traits Database ver 1. Environmental Data Initiative. https://doi.org/10.6073/pasta/5b3a6d80a1c6d3121a2196cb40838849 (Accessed 2022-05-22).

Fernández, R.J. and Reynolds, J.F., 2000. Potential growth and drought tolerance of eight desert grasses: lack of a trade-off?. Oecologia, 123(1), pp.90-98.

Fernández, R.J., Wang, M. and Reynolds, J.F., 2002. Do morphological changes mediate plant responses to water stress? A steady‐state experiment with two C4 grasses. New Phytologist, 155(1), pp.79-88.

Fim, Jennifer; Nguyen, Huong; Schütz, Martin; Risch, Anita C. (2019), Data from: Leaf trait variability between and within subalpine grassland species differs depending on site conditions and herbivory, v2, Dryad, Dataset, <https://doi.org/10.5061/dryad.n41d2dv>

Firn, J., Prober, S.M. and Buckley, Y.M., 2012. Plastic traits of an exotic grass contribute to its abundance but are not always favourable. PloS one, 7(4), p.e35870.

Firn, J., McGree, J.M., Harvey, E., Flores-Moreno, H., Schütz, M., Buckley, Y.M., Borer, E.T., Seabloom, E.W., La Pierre, K.J., MacDougall, A.M. and Prober, S.M., 2019. Leaf nutrients, not specific leaf area, are consistent indicators of elevated nutrient inputs. *Nature Ecology & Evolution*, *3*(3), pp.400-406.

Fitter, A.H. and Peat, H.J., 1994. The ecological flora database. Journal of Ecology, 82(2), pp.415-425.

Fonseca, C.R., Overton, J.M., Collins, B. and Westoby, M., 2000. Shifts in trait‐combinations along rainfall and phosphorus gradients. Journal of Ecology, 88(6), pp.964-977.

Forrestel, E.J., Donoghue, M.J., Edwards, E.J., Jetz, W., du Toit, J.C. and Smith, M.D., 2017. Different clades and traits yield similar grassland functional responses. Proceedings of the National Academy of Sciences, 114(4), pp.705-710.

Fort, F., Cruz, P. and Jouany, C., 2014. Hierarchy of root functional trait values and plasticity drive early‐stage competition for water and phosphorus among grasses. Functional Ecology, 28(4), pp.1030-1040.

Fort, F., Jouany, C. and Cruz, P., 2012. Root and leaf functional trait relations in Poaceae species: implications of differing resource-acquisition strategies. Journal of Plant Ecology, 6(3), pp.211-219.

Frenette‐Dussault, C., Shipley, B., Léger, J.F., Meziane, D. and Hingrat, Y., 2012. Functional structure of an arid steppe plant community reveals similarities with Grime's C‐S‐R theory. Journal of Vegetation Science, 23(2), pp.208-222.

Freschet, G.T., Cornelissen, J.H., Van Logtestijn, R.S. and Aerts, R., 2010. Evidence of the ‘plant economics spectrum in a subarctic flora. Journal of Ecology, 98(2), pp.362-373.

Frutos Tena, Á.D., Navarro, T., Pueyo, Y. and Alados, C.L., 2015. Inferring resilience to fragmentation-induced changes in plant communities in a semi-arid Mediterranean ecosystem.

Fry, E.L., Power, S.A. and Manning, P., 2014. Trait‐based classification and manipulation of plant functional groups for biodiversity–ecosystem function experiments. Journal of Vegetation Science, 25(1), pp.248-261.

Funk, J.L. and Vitousek, P.M., 2007. Resource-use efficiency and plant invasion in low-resource systems. Nature, 446(7139), p.1079.

Funk, J.L., 2008. Differences in plasticity between invasive and native plants from a low resource environment. Journal of Ecology, 96(6), pp.1162-1173.

Fynn, R., Morris, C., Ward, D. and Kirkman, K., 2011. Trait–environment relations for dominant grasses in South African mesic grassland support a general leaf economic model. *Journal of Vegetation Science*, *22*(3), pp.528-540.

Gachet, S., Véla, E. and Tatoni, T., 2005. BASECO: a floristic and ecological database of Mediterranean French flora. Biodiversity & Conservation, 14(4), pp.1023-1034.

Garnier, E. and Laurent, G., 1994. Leaf anatomy, specific mass and water content in congeneric annual and perennial grass species. New Phytologist, 128(4), pp.725-736.

Garnier, E. and Vancaeyzeele, S., 1994. Carbon and nitrogen content of congeneric annual and perennial grass species: relationships with growth. Plant, Cell & Environment, 17(4), pp.399-407.

Garnier, E., Cordonnier, P., Guillerm, J.L. and Sonié, L., 1997. Specific leaf area and leaf nitrogen concentration in annual and perennial grass species growing in Mediterranean old-fields. Oecologia, 111(4), pp.490-498.

Garnier, E., Laurent, G., Bellmann, A., Debain, S., Berthelier, P., Ducout, B., Roumet, C. and Navas, M.L., 2001. Consistency of species ranking based on functional leaf traits. New phytologist, 152(1), pp.69-83.

Garnier, E., Salager, J.L., Laurent, G. and Sonié, L., 1999. Relationships between photosynthesis, nitrogen and leaf structure in 14 grass species and their dependence on the basis of expression. The New Phytologist, 143(1), pp.119-129.

Geng, Y., Ma, W., Wang, L., Baumann, F., Kühn, P., Scholten, T. and He, J.S., 2017. Linking above‐and belowground traits to soil and climate variables: an integrated database on C hina's grassland species. Ecology, 98(5), pp.1471-1471.

Giarrizzo, E., Burrascano, S., Chiti, T., de Bello, F., Lepš, J., Zavattero, L. and Blasi, C., 2017. Re‐visiting historical semi‐natural grasslands in the Apennines to assess patterns of changes in species composition and functional traits. Applied Vegetation Science, 20(2), pp.247-258.

Grassein, F., Lemauviel-Lavenant, S., Lavorel, S., Bahn, M., Bardgett, R.D., Desclos-Theveniau, M. and Laîné, P., 2014. Relationships between functional traits and inorganic nitrogen acquisition among eight contrasting European grass species. Annals of botany, 115(1), pp.107-115.

Green, W., 2009. USDA PLANTS Compilation, version 1, 09‐02‐02.

Griffith, D. and Anderson, T.M., 2013. Responses of African grasses in the genus Sporobolus to defoliation and sodium stress: tradeoffs, cross-tolerance, or independent responses?. Plants, 2(4), pp.712-725.

Griffith, D.M., Quigley, K.M. and Anderson, T.M., 2016. Leaf thickness controls variation in leaf mass per area (LMA) among grazing-adapted grasses in Serengeti. Oecologia, 181(4), pp.1035-1040.

Grootemaat, S., Wright, I.J., van Bodegom, P.M., Cornelissen, J.H. and Cornwell, W.K., 2015. Burn or rot: leaf traits explain why flammability and decomposability are decoupled across species. Functional Ecology, 29(11), pp.1486-1497.

Gulías, J., Flexas, J., Mus, M., Cifre, J., Lefi, E. and Medrano, H., 2003. Relationship between maximum leaf photosynthesis, nitrogen content and specific leaf area in Balearic endemic and non‐endemic Mediterranean species. Annals of Botany, 92(2), pp.215-222.

Guy, A.L., Mischkolz, J.M. and Lamb, E.G., 2013. Limited effects of simulated acidic deposition on seedling survivorship and root morphology of endemic plant taxa of the Athabasca Sand Dunes in well-watered greenhouse trials. Botany, 91(3), pp.176-181.

Han, W., Chen, Y., Zhao, F.J., Tang, L., Jiang, R. and Zhang, F., 2012. Floral, climatic and soil pH controls on leaf ash content in China's terrestrial plants. Global Ecology and Biogeography, 21(3), pp.376-382.

Han, W., Fang, J., Guo, D. and Zhang, Y., 2005. Leaf nitrogen and phosphorus stoichiometry across 753 terrestrial plant species in China. New Phytologist, 168(2), pp.377-385.

He, J.S., Wang, Z., Wang, X., Schmid, B., Zuo, W., Zhou, M., Zheng, C., Wang, M. and Fang, J., 2006. A test of the generality of leaf trait relationships on the Tibetan Plateau. New Phytologist, 170(4), pp.835-848.

Herz, K., Dietz, S., Haider, S., Jandt, U., Scheel, D. and Bruelheide, H., 2017. Drivers of intraspecific trait variation of grass and forb species in German meadows and pastures. Journal of Vegetation Science, 28(4), pp.705-716.

Herz, K., Dietz, S., Haider, S., Jandt, U., Scheel, D. and Bruelheide, H., 2017. Drivers of intraspecific trait variation of grass and forb species in German meadows and pastures. Journal of Vegetation Science, 28(4), pp.705-716.

Hill, M.O., Preston, C.D. and Roy, D.B., 2004. PLANTATT-attributes of British and Irish plants: status, size, life history, geography and habitats. Centre for Ecology & Hydrology.

Hunt, R. and Cornelissen, J.H.C., 1997. Components of relative growth rate and their interrelations in 59 temperate plant species. The New Phytologist, 135(3), pp.395-417.

Iversen, C.M., McCormack, M.L., Powell, A.S., Blackwood, C.B., Freschet, G.T., Kattge, J., Roumet, C., Stover, D.B., Soudzilovskaia, N.A., Valverde‐Barrantes, O.J. and van Bodegom, P.M., 2017. A global fine‐root ecology database to address below‐ground challenges in plant ecology. New Phytologist, 215(1), pp.15-26.

Ivory, D.A. and Whiteman, P.C., 1978. Effect of temperature on growth of five subtropical grasses. I. Effect of day and night temperature on growth and morphological development. *Functional Plant Biology*, *5*(2), pp.131-148.

James, J.J., 2008. Leaf nitrogen productivity as a mechanism driving the success of invasive annual grasses under low and high nitrogen supply. Journal of Arid Environments, 72(10), pp.1775-1784.

Jardine, Emma et al. (2020), Data from: The global distribution of grass functional traits within grassy biomes, Dryad, Dataset, <https://doi.org/10.5061/dryad.s4mw6m938>

Jaurena, M., Lezama, F. and Cruz, P., 2012. Perennial grasses traits as functional markers of grazing intensity in basaltic grasslands of Uruguay. *Chilean Journal of Agricultural Research*, *72*(4), pp.541-549.

Juneau, K.J. and Tarasoff, C.S., 2012. Leaf area and water content changes after permanent and temporary storage. PLoS One, 7(8), p.e42604.

Kattge, J., Knorr, W., Raddatz, T. and Wirth, C., 2009. Quantifying photosynthetic capacity and its relationship to leaf nitrogen content for global‐scale terrestrial biosphere models. Global Change Biology, 15(4), pp.976-991.

Kazakou, E., Vile, D., Shipley, B., Gallet, C. and Garnier, E., 2006. Co‐variations in litter decomposition, leaf traits and plant growth in species from a Mediterranean old‐field succession. Functional Ecology, 20(1), pp.21-30.

Kerkhoff, A.J., Fagan, W.F., Elser, J.J. and Enquist, B.J., 2006. Phylogenetic and growth form variation in the scaling of nitrogen and phosphorus in the seed plants. The American Naturalist, 168(4), pp.E103-E122.

Kew, R.B.G., 2008. Seed information database (SID). Version 7.1.

Khalil, M.I., Gibson, D.J. and Baer, S.G., 2019. Functional response of subordinate species to intraspecific trait variability within dominant species. Journal of Ecology, 107(5), pp.2040-2053.

Kichenin, E., Wardle, D.A., Peltzer, D.A., Morse, C.W. and Freschet, G.T., 2013. Contrasting effects of plant inter‐and intraspecific variation on community‐level trait measures along an environmental gradient. Functional Ecology, 27(5), pp.1254-1261.

Kleyer, M., Bekker, R.M., Knevel, I.C., Bakker, J.P., Thompson, K., Sonnenschein, M., Poschlod, P., Van Groenendael, J.M., Klimeš, L., Klimešová, J. and Klotz, S.R.G.M., 2008. The LEDA Traitbase: a database of life‐history traits of the Northwest European flora. Journal of ecology, 96(6), pp.1266-1274.

Kleyer, M., Trinogga, J., Cebrián‐Piqueras, M.A., Trenkamp, A., Fløjgaard, C., Ejrnæs, R., Bouma, T.J., Minden, V., Maier, M., Mantilla‐Contreras, J. and Albach, D.C., 2019. Trait correlation network analysis identifies biomass allocation traits and stem specific length as hub traits in herbaceous perennial plants. *Journal of Ecology*, *107*(2), pp.829-842.

Kraft, N.J., Valencia, R. and Ackerly, D.D., 2008. Functional traits and niche-based tree community assembly in an Amazonian forest. Science, 322(5901), pp.580-582.

Kühn, I., Durka, W. and Klotz, S., 2004. BiolFlor: a new plant-trait database as a tool for plant invasion ecology. Diversity and Distributions, 10(5/6), pp.363-365.

Lachaise, T., Bergmann, J., Rillig, M. and van Kleunen, M., 2020. Below-and aboveground traits explain success of German grassland plants from plot to global scales. *Authorea Preprints*.

La Pierre, K.J. and Smith, M.D., 2015. Functional trait expression of grassland species shift with short-and long-term nutrient additions. Plant Ecology, 216(2), pp.307-318.

Larson, J.E., Sheley, R.L., Hardegree, S.P., Doescher, P.S. and James, J.J., 2015. Seed and seedling traits affecting critical life stage transitions and recruitment outcomes in dryland grasses. Journal of Applied Ecology, 52(1), pp.199-209.

Larson, J.E., Sheley, R.L., Hardegree, S.P., Doescher, P.S. and James, J.J., 2016. Do key dimensions of seed and seedling functional trait variation capture variation in recruitment probability?. Oecologia, 181(1), pp.39-53.

Laughlin, D.C., Leppert, J.J., Moore, M.M. and Sieg, C.H., 2010. A multi‐trait test of the leaf‐height‐seed plant strategy scheme with 133 species from a pine forest flora. Functional Ecology, 24(3), pp.493-501.

Laughlin, D.C., Leppert, J.J., Moore, M.M. and Sieg, C.H., 2010. A multi‐trait test of the leaf‐height‐seed plant strategy scheme with 133 species from a pine forest flora. Functional Ecology, 24(3), pp.493-501.

Lhotsky, B., Csecserits, A., Kovács, B. and Botta-Dukát, Z., 2016. New plant trait records of the Hungarian flora. Acta Botanica Hungarica, 58(3-4), pp.397-400.

Li, W., Xu, F., Zheng, S., Taube, F. and Bai, Y., 2017. Patterns and thresholds of grazing‐induced changes in community structure and ecosystem functioning: Species‐level responses and the critical role of species traits. Journal of Applied Ecology, 54(3), pp.963-975.

Li, Y. and Shipley, B., 2018. Community divergence and convergence along experimental gradients of stress and disturbance. Ecology, 99(4), pp.775-781.

Lin, Y.S., Medlyn, B.E., Duursma, R.A., Prentice, I.C., Wang, H., Baig, S., Eamus, D., De Dios, V.R., Mitchell, P., Ellsworth, D.S. and De Beeck, M.O., 2015. Optimal stomatal behaviour around the world. Nature Climate Change, 5(5), pp.459-464.

Lisner, A., Pärtel, M., Helm, A., Prangel, E. and Lepš, J., 2021. Traits as determinants of species abundance in a grassland community. *Journal of Vegetation Science*, *32*(3), p.e13041.

Liu, G., Freschet, G.T., Pan, X., Cornelissen, J.H., Li, Y. and Dong, M., 2010. Coordinated variation in leaf and root traits across multiple spatial scales in Chinese semi‐arid and arid ecosystems. New Phytologist, 188(2), pp.543-553.

Liu, G., Wang, L., Jiang, L., Pan, X., Huang, Z., Dong, M. and Cornelissen, J.H., 2018. Specific leaf area predicts dryland litter decomposition via two mechanisms. Journal of Ecology, 106(1), pp.218-229.

Liu, Guofang et al. (2018), Data from: Specific leaf area predicts dryland litter decomposition via two mechanisms, Dryad, Dataset, <https://doi.org/10.5061/dryad.60ns6>

Louault, F., Pillar, V.D., Aufrere, J., Garnier, E. and Soussana, J.F., 2005. Plant traits and functional types in response to reduced disturbance in a semi‐natural grassland. Journal of vegetation Science, 16(2), pp.151-160.

Loveys, B.R., Atkinson, L.J., Sherlock, D.J., Roberts, R.L., Fitter, A.H. and Atkin, O.K., 2003. Thermal acclimation of leaf and root respiration: an investigation comparing inherently fast‐and slow‐growing plant species. Global Change Biology, 9(6), pp.895-910.

Lundholm, J., Tran, S. and Gebert, L., 2015. Plant functional traits predict green roof ecosystem services. Environmental science & technology, 49(4), pp.2366-2374.

Maire, V., Gross, N., Pontes, L.D.S., Picon‐Cochard, C. and Soussana, J.F., 2009. Trade‐off between root nitrogen acquisition and shoot nitrogen utilization across 13 co‐occurring pasture grass species. Functional Ecology, 23(4), pp.668-679.

Maire, V., Wright, I.J., Prentice, I.C., Batjes, N.H., Bhaskar, R., van Bodegom, P.M., Cornwell, W.K., Ellsworth, D., Niinemets, Ü., Ordonez, A. and Reich, P.B., 2015. Global effects of soil and climate on leaf photosynthetic traits and rates. Global Ecology and Biogeography, 24(6), pp.706-717.

Martin, A.R., Hale, C.E., Cerabolini, B.E., Cornelissen, J.H., Craine, J., Gough, W.A., Kattge, J. and Tirona, C.K., 2018. Inter-and intraspecific variation in leaf economic traits in wheat and maize. AoB Plants, 10(1), p.ply006.

McJannet, C.L., Keddy, P.A. and Pick, F.R., 1995. Nitrogen and phosphorus tissue concentrations in 41 wetland plants: a comparison across habitats and functional groups. Functional Ecology, pp.231-238.

Meers, T.L., Kasel, S., Bell, T.L. and Enright, N.J., 2010. Conversion of native forest to exotic Pinus radiata plantation: Response of understorey plant composition using a plant functional trait approach. Forest Ecology and Management, 259(3), pp.399-409.

Mencuccini, M., 2003. The ecological significance of long‐distance water transport: short‐term regulation, long‐term acclimation and the hydraulic costs of stature across plant life forms. Plant, Cell & Environment, 26(1), pp.163-182.

Meziane, D. and Shipley, B., 1999. Interacting determinants of specific leaf area in 22 herbaceous species: effects of irradiance and nutrient availability. Plant, Cell & Environment, 22(5), pp.447-459.

Meziane, D. and Shipley, B., 2001. Direct and indirect relationships between specific leaf area, leaf nitrogen and leaf gas exchange. Effects of irradiance and nutrient supply. Annals of botany, 88(5), pp.915-927.

Milla, R. and Reich, P.B., 2011. Multi-trait interactions, not phylogeny, fine-tune leaf size reduction with increasing altitude. Annals of botany, 107(3), pp.455-465.

Miller, J.E., Ives, A.R., Harrison, S.P. and Damschen, E.I., 2018. Early‐and late‐flowering guilds respond differently to landscape spatial structure. Journal of Ecology, 106(3), pp.1033-1045.

Minden, V. and Kleyer, M., 2011. Testing the effect–response framework: key response and effect traits determining above‐ground biomass of salt marshes. Journal of Vegetation Science, 22(3), pp.387-401.

Minden, V. and Kleyer, M., 2014. Internal and external regulation of plant organ stoichiometry. Plant Biology, 16(5), pp.897-907.

Minden, V. and Kleyer, M., 2015. Ecosystem multifunctionality of coastal marshes is determined by key plant traits. Journal of vegetation science, 26(4), pp.651-662.

Minden, V., Andratschke, S., Spalke, J., Timmermann, H. and Kleyer, M., 2012. Plant trait–environment relationships in salt marshes: Deviations from predictions by ecological concepts. Perspectives in Plant Ecology, Evolution and Systematics, 14(3), pp.183-192.

Minden, Vanessa; olde Venterink, Harry (2019), Data from: Plant traits and species interactions along gradients of N, P and K availabilities, v2, Dryad, Dataset, https://doi.org/10.5061/dryad.77447g3

Moles, A.T., Falster, D.S., Leishman, M.R. and Westoby, M., 2004. Small‐seeded species produce more seeds per square metre of canopy per year, but not per individual per lifetime. Journal of ecology, 92(3), pp.384-396.

Montti, L., Villagra, M., Campanello, P.I., Gatti, M.G. and Goldstein, G., 2014. Functional traits enhance invasiveness of bamboos over co-occurring tree saplings in the semideciduous Atlantic Forest. Acta Oecologica, 54, pp.36-44.

Moretti, M. and Legg, C., 2009. Combining plant and animal traits to assess community functional responses to disturbance. Ecography, 32(2), pp.299-309.

Msadek, J. and Tarhouni, M., 2022. Biodiversity Assessment and Conservation of Threatened Plant Species Belonging to the Unique Steppe with Trees in Tunisian Drylands.

Mudrak, Ondrej; Dolezal, Jiri; Vítová, Alena; Leps, Jan (2019), Data from: Variation in plant functional traits is best explained by the species identity: stability of trait based species ranking across meadow management regimes, Dryad, Dataset, <https://doi.org/10.5061/dryad.v870905>

Onoda, Y., Wright, I.J., Evans, J.R., Hikosaka, K., Kitajima, K., Niinemets, Ü., Poorter, H., Tosens, T. and Westoby, M., 2017. Physiological and structural tradeoffs underlying the leaf economics spectrum. New Phytologist, 214(4), pp.1447-1463.

Ordonez, J.C., van Bodegom, P.M., Witte, J.P.M., Bartholomeus, R.P., van Hal, J.R. and Aerts, R., 2009. Plant strategies in relation to resource supply in mesic to wet environments: does theory mirror nature?. The American Naturalist, 175(2), pp.225-239.

Orwin, K.H., Buckland, S.M., Johnson, D., Turner, B.L., Smart, S., Oakley, S. and Bardgett, R.D., 2010. Linkages of plant traits to soil properties and the functioning of temperate grassland. Journal of Ecology, 98(5), pp.1074-1083.

Orwin, K.H., Mason, N.W., Jordan, O.M., Lambie, S.M., Stevenson, B.A. and Mudge, P.L., 2018. Season and dominant species effects on plant trait‐ecosystem function relationships in intensively grazed grassland. Journal of Applied Ecology, 55(1), pp.236-245.

Öster, M. and Eriksson, O., 2012. Recruitment in species-rich grasslands: the effects of functional traits and propagule pressure. Journal of Plant Ecology, 5(3), pp.260-269.

Oyarzabal, M., Paruelo, J.M., del Pino, F., Oesterheld, M. and Lauenroth, W.K., 2008. Trait differences between grass species along a climatic gradient in South and North America. Journal of Vegetation Science, 19(2), pp.183-192.

Padullés Cubino, J., Buckley, H.L., Day, N.J., Pieper, R. and Curran, T.J., 2018. Community‐level flammability declines over 25 years of plant invasion in grasslands. Journal of Ecology, 106(4), pp.1582-1594.

Paula, S., Arianoutsou, M., Kazanis, D., Tavsanoglu, Ç., Lloret, F., Buhk, C., Ojeda, F., Luna, B., Moreno, J.M., Rodrigo, A. and Espelta, J.M., 2009. Fire‐related traits for plant species of the Mediterranean Basin: Ecological Archives E090‐094. Ecology, 90(5), pp.1420-1420.

Peco, B., de Pablos, I., Traba, J. and Levassor, C., 2005. The effect of grazing abandonment on species composition and functional traits: the case of dehesa grasslands. Basic and applied Ecology, 6(2), pp.175-183.

Pescador, D.S., de Bello, F., Valladares, F. and Escudero, A., 2015. Plant trait variation along an altitudinal gradient in mediterranean high mountain grasslands: controlling the species turnover effect. PLoS One, 10(3).

Pontes, L.D.S., Soussana, J.F., Louault, F., Andueza, D. and Carrere, P., 2007. Leaf traits affect the above‐ground productivity and quality of pasture grasses. Functional Ecology, 21(5), pp.844-853.

Poorter, H. and De Jong, R.O.B., 1999. A comparison of specific leaf area, chemical composition and leaf construction costs of field plants from 15 habitats differing in productivity. The New Phytologist, 143(1), pp.163-176.

Prentice, I.C., Meng, T., Wang, H., Harrison, S.P., Ni, J. and Wang, G., 2011. Evidence of a universal scaling relationship for leaf CO2 drawdown along an aridity gradient. New Phytologist, 190(1), pp.169-180.

Pyankov, V.I., Kondratchuk, A.V. and Shipley, B., 1999. Leaf structure and specific leaf mass: the alpine desert plants of the Eastern Pamirs, Tadjikistan. The New Phytologist, 143(1), pp.131-142.

Pyankov, V.I., Kondratchuk, A.V. and Shipley, B., 1999. Leaf structure and specific leaf mass: the alpine desert plants of the Eastern Pamirs, Tadjikistan. The New Phytologist, 143(1), pp.131-142.

Quested, H.M., Cornelissen, J.H.C., Press, M.C., Callaghan, T.V., Aerts, R., Trosien, F., Riemann, P., Gwynn-Jones, D., Kondratchuk, A. and Jonasson, S.E., 2003. Decomposition of sub‐arctic plants with differing nitrogen economies: a functional role for hemiparasites. Ecology, 84(12), pp.3209-3221.

Rada, F., Sarmiento, L. and García-Varela, S., 2021. Plant functional traits along an old-field succession in the high tropical andes. *Acta Oecologica*, *111*, p.103738.

Reich, P.B. and Oleksyn, J., 2004. Global patterns of plant leaf N and P in relation to temperature and latitude. Proceedings of the National Academy of Sciences, 101(30), pp.11001-11006.

Reich, P.B., Buschena, C., Tjoelker, M.G., Wrage, K., Knops, J., Tilman, D. and Machado, J.L., 2003. Variation in growth rate and ecophysiology among 34 grassland and savanna species under contrasting N supply: a test of functional group differences. New Phytologist, 157(3), pp.617-631.

Rogers, A., Serbin, S.P., Ely, K.S., Sloan, V.L. and Wullschleger, S.D., 2017. Terrestrial biosphere models underestimate photosynthetic capacity and CO2 assimilation in the Arctic. New Phytologist, 216(4), pp.1090-1103.

Rolo, V., López-Díaz, M.L. and Moreno, G., 2012. Shrubs affect soil nutrients availability with contrasting consequences for pasture understory and tree overstory production and nutrient status in Mediterranean grazed open woodlands. Nutrient Cycling in Agroecosystems, 93(1), pp.89-102.

Roumet, C., Lafont, F., Sari, M., Warembourg, F. and Garnier, E., 2008. Root traits and taxonomic affiliation of nine herbaceous species grown in glasshouse conditions. Plant and Soil, 312(1-2), pp.69-83.

Roumet, C., Urcelay, C. and Díaz, S., 2006. Suites of root traits differ between annual and perennial species growing in the field. New phytologist, 170(2), pp.357-368.

Ryser, P. and Urbas, P., 2000. Ecological significance of leaf life span among Central European grass species. Oikos, 91(1), pp.41-50.

Ryser, P. and Wahl, S., 2001. Interspecific variation in RGR and the underlying traits among 24 grass species grown in full daylight. Plant Biology, 3(04), pp.426-436.

Sandel, B., Corbin, J.D. and Krupa, M., 2011. Using plant functional traits to guide restoration: a case study in California coastal grassland. Ecosphere, 2(2), pp.1-16.

Sandel, B., Pavelka, C., Hayashi, T., Charles, L., Funk, J., Halliday, F.W., Kandlikar, G.S., Kleinhesselink, A.R., Kraft, N.J., Larios, L. and Madsen‐McQueen, T., 2021. Predicting intraspecific trait variation among California's grasses. *Journal of Ecology*, *109*(7), pp.2662-2677.

Schöb, C., Macek, P., Pistón, N., Kikvidze, Z. and Pugnaire, F.I., 2017. A trait‐based approach to understand the consequences of specific plant interactions for community structure. Journal of Vegetation Science, 28(4), pp.696-704.

Schroeder‐Georgi, T., Wirth, C., Nadrowski, K., Meyer, S.T., Mommer, L. and Weigelt, A., 2016. From pots to plots: hierarchical trait‐based prediction of plant performance in a mesic grassland. Journal of Ecology, 104(1), pp.206-218.

Schultz, R.E., 2010. Plant diversity and community composition effects on carbon cycling and nitrogen partitioning in freshwater wetlands (Doctoral dissertation, The Ohio State University).

Sheremet’ev, S.N., 2005. Herbs on the soil moisture gradient (water relations and the structural-functional organization). KMK, Moscow, 271.

Shiodera, S., Rahajoe, J.S. and Kohyama, T., 2008. Variation in longevity and traits of leaves among co-occurring understorey plants in a tropical montane forest. Journal of tropical Ecology, 24(2), pp.121-133.

Shipley, B. and Lechowicz, M.J., 2000. The functional co-ordination of leaf morphology, nitrogen concentration, and gas exchange in40 wetland species. Ecoscience, 7(2), pp.183-194.

Shipley, B. and Parent, M., 1991. Germination responses of 64 wetland species in relation to seed size, minimum time to reproduction and seedling relative growth rate. Functional Ecology, pp.111-118.

Shipley, B. and Vu, T.T., 2002. Dry matter content as a measure of dry matter concentration in plants and their parts. New Phytologist, 153(2), pp.359-364.

Shipley, B., 1995. Structured interspecific determinants of specific leaf area in 34 species of herbaceous angiosperms. Functional Ecology, pp.312-319.

Shipley, B., 2002. Trade‐offs between net assimilation rate and specific leaf area in determining relative growth rate: relationship with daily irradiance. Functional ecology, 16(5), pp.682-689.

Siebenkäs, A., Schumacher, J. and Roscher, C., 2015. Phenotypic plasticity to light and nutrient availability alters functional trait ranking across eight perennial grassland species. AoB Plants, 7.

Siefert, A., Fridley, J.D. and Ritchie, M.E., 2014. Community functional responses to soil and climate at multiple spatial scales: when does intraspecific variation matter?. PLoS one, 9(10).

Simpson, Kimberley et al. (2021), Data from: C4 photosynthesis and the economic spectra of leaf and root traits independently influence growth rates in grasses, Dryad, Dataset, <https://doi.org/10.5061/dryad.xwdbrv1b1>

Smith, M.D. and Knapp, A.K., 2001. Physiological and morphological traits of exotic, invasive exotic, and native plant species in tallgrass prairie. International Journal of Plant Sciences, 162(4), pp.785-792.

Smith, N.G. and Dukes, J.S., 2017. 379201. LCE: leaf carbon exchange data set for tropical, temperate, and boreal species of North and Central America. Ecology, 98(11), p.2978.

Smith, S.D., Strain, B.R. and Sharkey, T.D., 1987. Effects of CO 2 enrichment on four Great Basin grasses. Functional Ecology, pp.139-143.

Smith, S.W., Woodin, S.J., Pakeman, R.J., Johnson, D. and Van Der Wal, R., 2014. Root traits predict decomposition across a landscape‐scale grazing experiment. New Phytologist, 203(3), pp.851-862.

Solofondranohatra, C.L., Vorontsova, M.S., Dewhirst, R.A., Belcher, C.M., Cable, S., Jeannoda, V. and Lehmann, C.E., 2021. Shade alters the growth and architecture of tropical grasses by reducing root biomass. *Biotropica*, *53*(4), pp.1052-1062.

Soudzilovskaia, N.A., Elumeeva, T.G., Onipchenko, V.G., Shidakov, I.I., Salpagarova, F.S., Khubiev, A.B., Tekeev, D.K. and Cornelissen, J.H., 2013. Functional traits predict relationship between plant abundance dynamic and long-term climate warming. Proceedings of the National Academy of Sciences, 110(45), pp.18180-18184.

Spasojevic, M.J. and Suding, K.N., 2012. Inferring community assembly mechanisms from functional diversity patterns: the importance of multiple assembly processes. Journal of Ecology, 100(3), pp.652-661.

Spasojevic, M., S. Weber, and Niwot Ridge LTER. 2020. Niwot plant functional traits from 2008 to 2018 ver 2. Environmental Data Initiative. https://doi.org/10.6073/pasta/21a28cfb55afeae56a1ef4ef9c8f5b6a (Accessed 2022-05-22).

Stanisci, A., Bricca, A., Calabrese, V., Cutini, M., Pauli, H., Steinbauer, K. and Carranza, M.L., 2020. Functional composition and diversity of leaf traits in subalpine versus alpine vegetation in the Apennines. *AoB Plants*, *12*(2), p.plaa004.

Stromberg, J.C., 2013. Root patterns and hydrogeomorphic niches of riparian plants in the American Southwest. Journal of Arid Environments, 94, pp.1-9.

Sugiyama, S., 2005. Developmental basis of interspecific differences in leaf size and specific leaf area among C3 grass species. Functional Ecology, 19(6), pp.916-924.

Sweeney, C.J., de Vries, F.T., van Dongen, B.E. and Bardgett, R.D., 2021. Root traits explain rhizosphere fungal community composition among temperate grassland plant species. *New Phytologist*, *229*(3), pp.1492-1507.

Takkis, K., 2014. Changes in plant species richness and population performance in response to habitat loss and fragmentation (Doctoral dissertation).

Targetti, S., Messeri, A., Staglianò, N. and Argenti, G., 2013. Leaf functional traits for the assessment of succession following management in semi‐natural grasslands: a case study in the North Apennines, Italy. Applied Vegetation Science, 16(2), pp.325-332.

Taylor, S.H., Hulme, S.P., Rees, M., Ripley, B.S., Ian Woodward, F. and Osborne, C.P., 2010. Ecophysiological traits in C3 and C4 grasses: a phylogenetically controlled screening experiment. New Phytologist, 185(3), pp.780-791.

Thorne, M.A. and Frank, D.A., 2009. The effects of clipping and soil moisture on leaf and root morphology and root respiration in two temperate and two tropical grasses. Plant Ecology, 200(2), pp.205-215.

Tipping, C. and Murray, D.R., 1999. Effects of elevated atmospheric CO2 concentration on leaf anatomy and morphology in Panicum species representing different photosynthetic modes. International journal of plant sciences, 160(6), pp.1063-1073.

Tjoelker, M.G., Craine, J.M., Wedin, D., Reich, P.B. and Tilman, D., 2005. Linking leaf and root trait syndromes among 39 grassland and savannah species. New Phytologist, 167(2), pp.493-508.

Tribouillois, H., Fort, F., Cruz, P., Charles, R., Flores, O., Garnier, E. and Justes, E., 2015. A functional characterisation of a wide range of cover crop species: growth and nitrogen acquisition rates, leaf traits and ecological strategies. PLoS One, 10(3), p.e0122156.

Tucker, S.S., 2010. Morphological and physiological traits as indicators of drought tolerance in tallgrass prairie plants (Doctoral dissertation, Kansas State University).

Tucker, S.S., Craine, J.M. and Nippert, J.B., 2011. Physiological drought tolerance and the structuring of tallgrass prairie assemblages. Ecosphere, 2(4), pp.1-19.

Tuihnman, V. 2020. The future of South African grassland communities: An experimental analysis on plant trait responses to fire frequency and warming. University of Utrecht Master’s Thesis.

Van Arendonk, J.J.C.M., Niemann, G.J., Boon, J.J. and Lambers, H., 1997. Effects of nitrogen supply on the anatomy and chemical composition of leaves of four grass species belonging to the genus Poa, as determined by image‐processing analysis and pyrolysis–mass spectrometry. Plant, Cell & Environment, 20(7), pp.881-897.

Van der Plas, F. and Olff, H., 2014. Mesoherbivores affect grasshopper communities in a megaherbivore-dominated South African savannah. Oecologia, 175(2), pp.639-649.

Van der Plas, F., Howison, R.A., Mpanza, N., Cromsigt, J.P. and Olff, H., 2016. Different‐sized grazers have distinctive effects on plant functional composition of an A frican savannah. Journal of Ecology, 104(3), pp.864-875.

Van Staalduinen, M.A. and Anten, N.P., 2005. Differences in the compensatory growth of two co-occurring grass species in relation to water availability. Oecologia, 146(2), pp.190-199.

Vendramini, F., Díaz, S., Gurvich, D.E., Wilson, P.J., Thompson, K. and Hodgson, J.G., 2002. Leaf traits as indicators of resource‐use strategy in floras with succulent species. New Phytologist, 154(1), pp.147-157.

Vergutz, L., Manzoni, S., Porporato, A., Novais, R.F. and Jackson, R.B., 2012. A global database of carbon and nutrient concentrations of green and senesced leaves. ORNL DAAC.

Verhoeven, D., Buckley, H.L. and Curran, T.J., 2014. Functional traits of common New Zealand foredune species at New Brighton, Canterbury. New Zealand journal of botany, 52(4), pp.460-466.

Vile, D., 2005. Significations fonctionnelle et écologique des traits des espèces végétales: exemple dans une succession post-culturale méditerranéenne et généralisations (Doctoral dissertation, Montpellier 2).

Villar, R., Maranon, T., Quero, J.L., Panadero, P., Arenas, F. and Lambers, H., 2005. Variation in relative growth rate of 20 Aegilops species (Poaceae) in the field: the importance of net assimilation rate or specific leaf area depends on the time scale. Plant and soil, 272(1-2), pp.11-27.

Walker, A., Arand, I., Beckerman, A., Bown, H., Cernusak, L., Dang, Q., Domingues, T., Gu, L., Guo, S., Han, Q. and Kattge, J., 2014. A Global Data Set of Leaf Photosynthetic Rates, Leaf N and P, and Specific Leaf Area. ORNL DAAC.

Wang, J., Wang, Z., Zhang, X., Zhang, Y., Ran, C., Zhang, J., Chen, B. and Zhang, B., 2015. Response of Kobresia pygmaea and Stipa purpurea grassland communities in northern Tibet to nitrogen and phosphate addition. *Mountain Research and Development*, *35*(1), pp.78-86.

Welsh, M.E., Cronin, J.P. and Mitchell, C.E., 2016. The role of habitat filtering in the leaf economics spectrum and plant susceptibility to pathogen infection. Journal of Ecology, 104(6), pp.1768-1777.

Winkler, N., Weymann, W., Auge, H., Klotz, S., Finkenbein, P. and Heilmeier, H., 2015. Drought resistance of native pioneer species indicates potential suitability for restoration of post-mining areas. Web Ecology, 14(1), pp.65-74.

Wirth, C. and Lichstein, J.W., 2009. The imprint of species turnover on old-growth forest carbon balances-insights from a trait-based model of forest dynamics. In Old-growth forests (pp. 81-113). Springer, Berlin, Heidelberg.

Wright, I.J., Dong, N., Maire, V., Prentice, I.C., Westoby, M., Díaz, S., Gallagher, R.V., Jacobs, B.F., Kooyman, R., Law, E.A. and Leishman, M.R., 2017. Global climatic drivers of leaf size. Science, 357(6354), pp.917-921.

Wright, I.J., Reich, P.B., Westoby, M., Ackerly, D.D., Baruch, Z., Bongers, F., Cavender-Bares, J., Chapin, T., Cornelissen, J.H., Diemer, M. and Flexas, J., 2004. The worldwide leaf economics spectrum. Nature, 428(6985), pp.821-827.

Wright, J.P. and Sutton‐Grier, A., 2012. Does the leaf economic spectrum hold within local species pools across varying environmental conditions?. Functional Ecology, 26(6), pp.1390-1398.

Yu, Q., Elser, J.J., He, N., Wu, H., Chen, Q., Zhang, G. and Han, X., 2011. Stoichiometric homeostasis of vascular plants in the Inner Mongolia grassland. Oecologia, 166(1), pp.1-10.

Yue, X., Zuo, X., Yu, Q., Xu, C., Lv, P., Zhang, J., Knapp, A.K. and Smith, M.D., 2019. Response of plant functional traits of Leymus chinensis to extreme drought in Inner Mongolia grasslands. Plant Ecology, 220(2), pp.141-149.

Yulin, L.I., Johnson, D.A., Yongzhong, S.U., Jianyuan, C.U.I. and Zhang, T., 2005. Specific leaf area and leaf dry matter content of plants growing in sand dunes. Botanical Bulletin of Academia Sinica, 46.

Zheng, S. and Shangguan, Z., 2007. Spatial patterns of photosynthetic characteristics and leaf physical traits of plants in the Loess Plateau of China. Plant ecology, 191(2), pp.279-293.

Zheng, S., Lan, Z., Li, W., Shao, R., Shan, Y., Wan, H., Taube, F. and Bai, Y., 2011. Differential responses of plant functional trait to grazing between two contrasting dominant C3 and C4 species in a typical steppe of Inner Mongolia, China. Plant and Soil, 340(1-2), pp.141-155.

Zheng, S.X., Ren, H.Y., Lan, Z.C., Li, W.H., Wang, K.B. and Bai, Y.F., 2010. Effects of grazing on leaf traits and ecosystem functioning in Inner Mongolia grasslands: scaling from species to community. Biogeosciences, 7(3), pp.1117-1132.

Zheng, W., 1983. Silva Sinica: Volume 1–4.

Zirbel, C.R., Bassett, T., Grman, E. and Brudvig, L.A., 2017. Plant functional traits and environmental conditions shape community assembly and ecosystem functioning during restoration. Journal of Applied Ecology, 54(4), pp.1070-1079.
